# A new ion imprinted polymer based on Ru(III)-thiobarbituric acid complex for solid phase extraction of ruthenium(III) prior to its determination by ETAAS

**DOI:** 10.1007/s00604-014-1190-0

**Published:** 2014-02-22

**Authors:** Elżbieta Zambrzycka, Beata Godlewska-Żyłkiewicz

**Affiliations:** Institute of Chemistry, University of Bialystok, Hurtowa 1, 15-399 Bialystok, Poland

**Keywords:** Ruthenium, Separation, Preconcentration, Ion imprinted polymers, Environmental samples, Electrothermal atomic absorption spectrometry

## Abstract

A new ruthenium ion imprinted polymer was prepared from the Ru(III) 2-thiobarbituric acid complex (the template), methacrylic acid or acrylamide (the functional monomers), and ethylene glycol dimethacrylate (the cross-linking agent) using 2,2′-azobisisobutyronitrile as the radical initiator. The ion imprinted polymer was characterized and used as a selective sorbent for the solid phase extraction of Ru(III) ions. The effects of type of functional monomer, sample volume, solution pH and flow rate on the extraction efficiency were studied in the dynamic mode. Ru(III) ion was quantitatively retained on the sorbents in the pH range from 3.5 to 10, and can be eluted with 4 mol L^−1^ aqueous ammonia. The affinity of Ru(III) for the ion imprinted polymer based on the acrylamide monomer is weaker than that for the polymer based on the methacrylic acid monomer, which therefore was used in interference studies and in analytical applications. Following extraction of Ru(III) ions with the imprint and their subsequent elution from the polymer with aqueous ammonia, Ru(III) was detected by electrothermal atomic absorption spectrometry with a detection limit of 0.21 ng mL^−1^. The method was successfully applied to the determination of trace amounts of Ru(III) in water, waste, road dust and platinum ore (CRM SARM 76) with a reproducibility (expressed as RSD) below 6.4 %.

**Figure:**
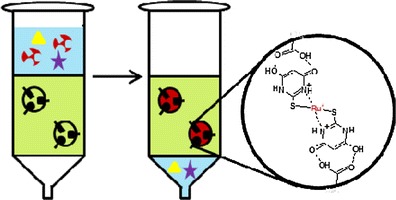
The new ion imprinted polymer was prepared and used for the separation of ruthenium from water and most complex environmental samples, such as road dust and platinum ore (CRM SARM 76) prior ETAAS determination.

The new ion imprinted polymer was prepared and used for the separation of ruthenium from water and most complex environmental samples, such as road dust and platinum ore (CRM SARM 76) prior ETAAS determination.

## Introduction

Ruthenium and its alloys are of commercial importance as they have widespread application in electronics, electrical and electrochemical industries. Above all, ruthenium and its complexes have been recognized as an efficient catalyst for a large number of reactions of commercial and environmental importance and exhibit classical catalyst characteristics in acidic as well as in alkaline media [1]. Ruthenium together with platinum is used as a bimetallic catalyst in electro-oxidation of methane in fuel cells [2]. Some ruthenium compounds possessing anticancer activity (e.g. NAMI-A, KP1019) are currently undergoing advanced preclinical testing [3]. Thus, the versatile use of ruthenium in different fields justifies the special attention in developing low cost, selective, sensitive and precise methods for its determination at trace levels.

Determination of ruthenium requires pretreatment of the sample ensuring quantitative conversion of ruthenium into soluble complexes, separation of the analyte from the interfering elements and preconcentration up to the level detected by the analytical technique employed. Solid phase extraction (SPE) based on anion-exchange resins is most often used for separation or preconcentration of ruthenium from the sample matrix [4]. Although the separation process using commercially available strong ion-exchangers is simple and efficient, these sorbents very often exhibit poor metal ion selectivity, as most of the platinum group metals are retained simultaneously. For the recovery of ruthenium from such sorbents either large volumes of concentrated mineral acids were used (e.g. 12 mL of 5 mol L^−1^ HNO_3_ + 5 mol L^−1^ HClO_4_ [5], 10 mL of concentrated HNO_3_ [6], 100 mL of 12 mol L^−1^ HCl [7]) or the resin was ashed and the residue was dissolved in acid before analysis [8]. Elution of ruthenium from polymers functionalized with chelating groups, such as polystyrene-divinylbenzene resin with thiosemicarbazide functional group [9], or polyacrylacylisothiourea chelating fibre [10] was also performed with aggressive reagents (9 mol L^−1^ HCl or 4 mol L^−1^ HCl + 1 % CS(NH_2_)_2_).

The research in the synthesis of new sorption materials is striving to achieve of molecule specific sorbents. Molecularly imprinted polymers are prepared by creating a three-dimensional polymeric matrix around a template molecule. After the template is removed, complementary cavities with respect to shape and functional groups remains in the polymeric structure. The recognition is based on the morphology or stereochemistry of cavities. For separation of metal ions the technology of synthesis of ion imprinted polymers (IIP) has been developed [11]. In the ion imprinting process, the selectivity of polymer is based on the specificity of a ligand, on the coordination geometry and coordination number of the ions, on their charge and size [12]. The separation of ruthenium by means of IIP has been recently proposed by our research group [13–15]. The prepared materials based on imprinted complexes of ruthenium(III) with thiosemicarbazide and acetaldehyde thiosemicarbazone [15], benzaldehyde thiosemicarbazone [13] and allyl acetoacetate [14] are characterized by good selectivity and stability. On that basis the selective and accurate procedures for ruthenium determination in samples of natural waters, municipal wastes, grass and hair have been developed. However, the fact that sorption of the analyte on IIP occurred in neutral or alkaline solutions (pH from 6.5 to 10) precluded their potential application for very complex environmental samples, such as geological materials or road dust (due to co-precipitation of analyte with hydroxides of metals present in sample matrix).

To develop a method suitable for the determination of ruthenium in such samples a new ion imprinted polymer was designed and synthesized. A 2-thiobarbituric acid (TBA) (2-thioxodihydro-4,6(1H,5H)-pyrimidinedione), containing a pyrimidine ring with electro donor atoms (N and S) and three mobile H atoms, was selected as a ligand complexing Ru(III) ion [16, 17]. In acidic solutions (pH 0.6–4) TBA forms a stable Ru(III)-TBA complex (1:2 molar ratio) through the coordination sulfur-metal bond [18]. The IIP was synthesized through bulk polymerization using Ru(III)-TBA complex as a template molecule, methacrylic acid or acrylamide as a functional monomer and ethylene glycol dimethacrylate as a cross-linking agent. The particles of IIP polymers were employed in SPE procedure for the separation of ruthenium from complex environmental samples (river water, sewage, road dust and platinum ore) before its determination by electrothermal atomic absorption spectrometry (ETAAS).

## Experimental

### Instrumentation

A Solaar M6 (Thermo Electron Corporation, UK, www.thermoscientific.com) atomic absorption spectrometer, equipped with an electrothermal atomizer, a Zeeman background correction system, and a ruthenium hollow cathode lamp (10 mA) (Thermo Scientific, USA, www.thermoscientific.com) were used for the determination of ruthenium. The integrated absorbance signal of ruthenium was measured at 349.9 nm with a spectral bandpass of 0.2 nm, using pyrolytically coated graphite tubes. The following optimized furnace heating program was used: drying at 110 ºC for 30 s, ashing at 1,200 ºC for 20 s, and atomization at 2,650 ºC for 3 s.

The FT-IR absorption spectra (4,000–500 cm^−1^) were recorded with KBr pellets using a Thermo Nicolet Magna IR 550 Series II (Nicolet, Japan, www.thermoscientific.com). A Surface Area and Porosity Analyser Gemini VII 2390 (Micromeritics, USA, www.micromeritics.com) was used for the determination of surface area by BET method. Nitrogen sorption analysis was carried out on approximately ~0.4 g portions of polymers degassed for 24 h at 80 °C.

An inoLab pH Level 1 (WTW, Germany, www.wtw.de) pH meter, equipped with a SenTix 21 electrode (WTW, Germany, www.wtw.de), was used for the pH measurements. A flow system used for the separation of ruthenium consisted of a peristaltic pump Minipuls 3 (Gilson, France, www.gilson.com), PTFE tubes with an i.d. of 0.8 mm, and glassy adsorption columns with an i.d. of 3.4 mm containing PTFE frits. The digestion of samples was performed in an ETHOS PLUS (Milestone, Italy, www.milestonesrl.com) microwave system.

### Reagents and materials

A ruthenium atomic spectroscopy standard solution in HCl (1 mg mL^−1^, Fluka, Switzerland, www.sigmaaldrich.com) was used. Sodium hydroxide (POCh, Poland, www.poch.com) solution (1 mol L^−1^) was used to adjust the pH of samples and standards. Acetic acid (80 %, POCh, Poland, www.poch.com) was used to remove interferents from columns. Ammonia (25 %, POCh, Poland, www.poch.com) and thiourea (puris p.a., Fluka, China, www.sigmaaldrich.com) were used as desorption agents. Nitric acid (69.5 %, Trace Select, Fluka, France, www.sigmaaldrich.com) and hydrochloric acid (37 %, fuming, Trace Select, Fluka, France, www.sigmaaldrich.com) were used for the digestion of samples.

Ruthenium(III) chloride hydrate (purum, 41 % Ru, Fluka, UK, www.sigmaaldrich.com), 2-thiobarbituric acid (TBA, Merck, Germany) and ethanol (99.8 %, POCh, Poland, www.poch.com) were used for the preparation of Ru(III) complexes. Ethylene glycol dimethacrylate (98 %, EGDMA, Sigma Aldrich, USA, www.sigmaaldrich.com), methacrylic acid (99 %, MMA, Sigma Aldrich, USA, www.sigmaaldrich.com), acrylamide (99.9 % pure, ACM, Bio-Rad Laboratories Headquarters, USA, www.bio-rad.com) and 2,2′-azobisisobutyronitrile (AIBN, Fluka, France, www.sigmaaldrich.com) were used for the synthesis of polymers. Methanol (99.8 %, POCh, Poland, www.poch.com) was used as a porogen. Ethyl acetate (POCh, Poland, www.poch.com) was used to remove the excess of polymerization reagents. Certified reference material - platinum ore (SARM 76, MINTEK, www.mintek.co.za) from the Merensky Reef area, South Africa, was used for accuracy studies.

Solutions of palladium(II), rhodium(III) and iron(III) chlorides, cobalt(II) and nickel(II) nitrates (SCP Science, Canada, www.scpscience.com), and platinum as hexachloroplatinic(IV) acid (30 %, POCh, Poland, www.poch.com) were used to study the matrix interference. All solutions were prepared in de-ionized water obtained from a Milli-Q water purification system (Millipore, USA, www.millipore.com).

### Synthesis of Ru(III)-2-thiobarbituric acid complex

The complex of Ru(III)-2-thiobarbituric acid (Ru(III)-TBA) (the template) was prepared according to the procedure described in [18]. Ruthenium(III) chloride hydrate (4.1 mg, 0.0198 mmol) was dissolved in 5.5 mL mixture of ethanol and concentrated HCl (1 + 1), next 1.6 mL of aqueous solution of TBA (7.1 mg, 0.0493 mmol TBA) was added and the resulting solution was diluted with ethanol to volume of 13 mL. The mixture was heated for 60 min on a water-bath at temp. 75 °C. The dark brown residue of the formed complex was dried in a vacuum evaporator. FT-IR (KBr, cm^−1^): 3,178–3,128 (υ_N−H_)_,_ 1,714 (υ_C=O_)_,_ 1,559–1,535 (δ_N−H_, υ_C=C_, υ_C=O_, υ_C=N_), 1,420 (δ_N−H_), 1,347 (υ_C=N_, υ_C=S_), 1,298–1,273 (υ_C−N_, υ_C=S_), 1,250 (υ_C=N_, υ_C-S_), 933, 801 (δ_C−H_). The main IR spectra of TBA and Ru(III)-TBA complex in the solid state show similar bands. Small differences were observed in the absorption bands (υ_N-H_), (υ_C=S_) and (υ_C-S_). The absorption band (υ_N-H_) of the ligand at 3,110 cm^−1^ was shifted to 3,178 cm^−1^ in Ru(III)-TBA complex suggesting that the NH groups took part in the formation of a bond with Ru(III) ion. Moreover, the band present at 1,298 cm^−1^ in the TBA spectrum (assigned to the C=S stretching vibrations) was considerably weakened and shifted to 1,282 cm^−1^ in the Ru(III)-TBA complex spectrum, which indicates that the coordination of Ru(III) took place through the sulphur atom of the C=S group.

### Preparation of ruthenium(III) imprinted polymers and their characteristics

**Fig. 1:**
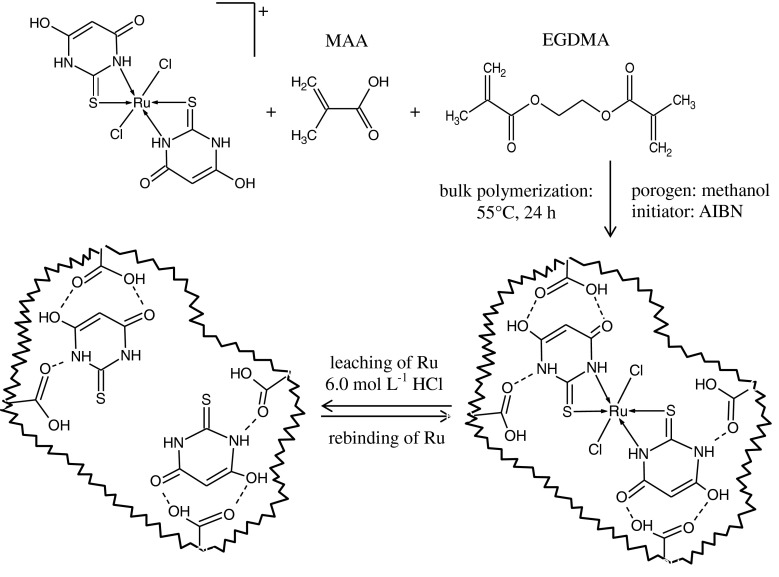
Schematic illustration of the imprinting process for preparation of the ruthenium imprinted polymer using methacrylic acid as functional monomer

The Ru(III) imprinted polymers were prepared by bulk polymerization technique according to the scheme presented in Fig. 1. The complex of Ru(III)-TBA containing 0.002 g (0.0197 mmol) of Ru(III) was dissolved in 4 mL of methanol and transferred into glassy polymerization ampoules. Then, methacrylic acid (MMA) (4.012 mmol) or acrylamide (ACM) (4.011 mmol), EGDMA (15.9 mmol) and AIBN (0.1 g) were added and the mixture was stirred until the solution was clear. The ampoules were purged with argon for 10 min to remove any dissolved oxygen, which could inhibit free radical polymerization. The reaction temperature was kept constant at 55 °C for 24 h. The resultant hard polymer monolith was crushed in a mortar and washed with ethyl acetate in order to remove the excess of reagents. The polymer was then dried, ground and sieved. The fraction of 100–150 μm in diameter was used as a column filling. Control polymers (CP) were synthesized in a similar way, but in the absence of Ru(III) ions. The imprinted Ru(III) ions were leached from individual portions of the polymers (0.4 g) by passing a 100 mL solution of 6.0 mol L^−1^ HCl.

The IIP and respective CP have similar IR spectra indicating the similarity in the backbone structure. FT-IR of Ru-TBA-MAA (KBr, cm^−1^): 3,565 (υ_O−H_)_,_ 2,992 (υ_C−H_)_,_ 1,732 (υ_C=O_), 1,256, 1,147 (υ_C−O_), 2,992, 2,959, 1,456, 1,392 and 754 (υ_C−H_); FT-IR of Ru-TBA-ACM polymer (KBr, cm^−1^): 3,448 (υ_N−H_), 2,956 (υ_C−H_), 1,729 (υ_C=O_), 1,261, 1,157 (υ_C−O_), 1,457, 1,389 and 754 (υ_C−H_). The characteristic bands of the polymeric matrix of MMA-EGDMA at 2,992, 2,959, 1,456, 1,392 and 754 cm^−1^ corresponding to the C-H stretching vibrations of the methylene groups (υ_C−H_) and at 1,732 cm^−1^ corresponding to the C=O stretching vibrations of the carbonyl group (υ_C=O_) were observed in the spectra of IIP and CP polymer. The FT-IR spectrum of the polymeric matrix of ACM-EGDMA showed characteristic bands at 3,448 cm^−1^ corresponding to the N-H stretching vibrations and at 1,729 cm^−1^ corresponding to the C=O stretching vibrations of the amide group. The characteristic strong absorption bands of the polymeric matrix obscure the weak bands originating from the imprinted Ru(III)-TBA complex.

**Fig. 2:**
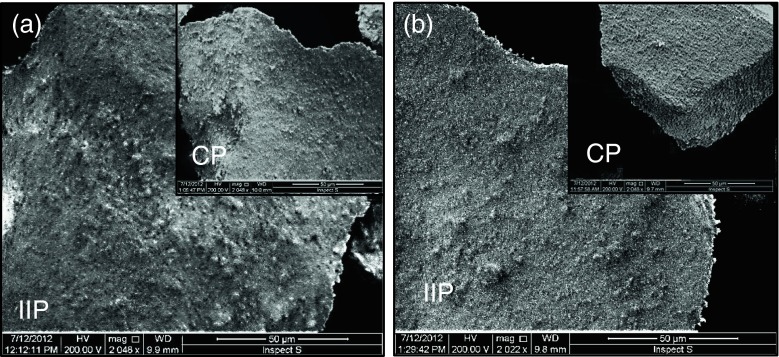
SEM images of the surface of the IIP and CP polymers (2,000-fold magnification) prepared using different functional monomers: **a** methacrylic acid, **b** acrylamide

The BET surface areas for the polymers prepared using methacrylic acid were 211.3 m^2^ g^−1^ for the CP and 232.9 m^2^ g^−1^ for the IIP. The BET surface areas for the polymers prepared using acrylamide were 121.3 m^2^ g^−1^ for the CP and 144.1 m^2^ g^−1^ for the IIP. Scanning electron microscopic images are presented in Fig. 2. The textural characteristics were examined at 2,000× magnification, revealing distinctive pattern on the imprinted surfaces, with more rough surface of the Ru-TBA-MAA polymer. The surface of both CP polymers displays the lack of comparable porous structure.

### Pre-treatment and analysis of environmental samples

Tap water, road runoff and municipal sewage were spiked with 50 ng mL^−1^ of ruthenium. River water was spiked with 1 and 50 ng mL^−1^ of ruthenium. After an overnight equilibration, the samples were filtered through PVDF filters (Whatman, 0.45 μm) and adjusted to pH 4.1 ± 0.1 with diluted NaOH. About 0.2 g of platinum ore (CRM SARM-76) and road dust collected from the main intersection located in the centre of Białystok (Poland) were weighed into Teflon vessels and leached with 8 mL of aqua regia using a microwave digestion system according to the procedure described in [19]. The 2 mL portions of solutions were transferred to quartz crucibles, evaporated with 2 mL of concentrated HCl near to dryness three times and diluted with water. The pH of such pretreated samples of road dust and platinum ore was adjusted to pH 3.5 using diluted NaOH directly before being loaded onto the column.

Glassy columns packed with 0.1 g of IIP were preconditioned by passing 4.5 mL of water. The samples (2–100 mL) adjusted to desired pH were passed through the column at a flow rate of 1.2 mL min^−1^. The elution of Ru(III) ions was accomplished by passing 2 mL of 4.0 mol L^−1^ ammonia solution through the column at a flow rate of 0.4 mL min^−1^. For the analysis of road dust and platinum ore the column was rinsed with 4 mL of 0.05 mol L^−1^ CH_3_COOH before elution step. The content of ruthenium in all solutions was analyzed by ETAAS.

## Results and discussion

### Optimization of ruthenium separation conditions

Our previous studies concerning the size of columns have shown that using longer and narrower columns resulted in improved efficiency of the retention of the analyte (up to 20 %) and better reproducibility of the separation process [13]. Therefore, in this work the glassy columns (110 mm × 3.4 mm i.d) filled with 0.1 g of polymer were used. The columns were preconditioned with water.

The effects of the sample pH and the rate of passing the sample solution through the columns, the kind of eluent, its flow rate and volume on the retention of Ru(III) ions were studied simultaneously on both IIP polymers. The retention efficiency was calculated as a ratio of the mass of Ru retained on the column to the initial mass of Ru loaded onto the column. It was observed that the efficiency of retention of analyte on Ru-TBA-MAA polymer was low (8–30 %) in acidic solutions (1 < pH ≤ 3), rapidly increased to 80 % in solutions of pH 3.5, and exceeded 90 % in solutions in the pH range from 4 to 10. The behavior of Ru-TBA-ACM polymer was very similar, however the efficiencies of ruthenium retention were lower by 3–10 % in whole studied pH range. In order to prevent precipitation of hydroxides of other metals present in the matrix of analyzed samples the sample pH was adjusted to pH 4.1 ± 0.1 in further studies. The efficiency of retention of Ru(III) on respective CP at the pH range of 3.5–5 was lower by 12–15 %. The flow rate of Ru(III) solutions in the range from 0.2 to 1.5 mL min^−1^ practically does not affect the efficiency of sorption on IIP (91.8–90.5 %). Thus, the flow rate of 1.2 ml L^−1^ was chosen for subsequent experiments. The similar flow rate (1.5 mL min^−1^) was used in the case of polymer based on Ru(III)-thiosemicarbazide complex [15], but much lower (0.6 mL min^−1^) in the case of polymer with imprinted Ru(III)-allyl acetoacetate complex [14]. This indicates that the Ru-TBA-MAA and Ru-TBA-ACM polymers are characterized by fast kinetics of sorption. Our previous studies [13–15] demonstrated that quantitative elution of Ru(III) from IIP could be obtained using acidic solutions of thiourea. Thus, solutions of different concentrations of thiourea and HCl (0.1–0.9 mol L^−1^ of thiourea and 0.1–0.5 mol L^−1^ of HCl) were tested as stripping agents. The efficiencies of elution of analyte, calculated as a ratio of the mass of Ru eluted from the column by a stripping agent to the mass of Ru retained on the column, with these eluents were not satisfactory (≤75 %), thus the ammonia solutions (0.2–5 mol L^−1^) were tested afterwards. The ruthenium elution from Ru-TBA-MAA polymer was rising with increasing concentrations of ammonia solutions (up to 87 % for 4 mol L^−1^ NH_3_·H_2_O). The effect of eluent flow rate on the efficiency of elution was studied in the range of 0.2–0.8 mL min^−1^. The highest efficiency of elution (87–94 %) was obtained at the flow rates of 0.2–0.4 mL min^−1^ with 2–3 mL of NH_3_·H_2_O. The efficiencies of ruthenium elution from the Ru-TBA-ACM polymer were higher by 3–6 % than from the Ru-TBA-MAA polymer.

It is worth stressing that the signal of ruthenium standard in 1–5 mol L^−1^ NH_3_·H_2_O solutions was about 40 % higher in comparison to its signal in water, but 20 % lower than in diluted solutions of HCl [15]. The other positive effect of the chosen eluent could be that ammonia complexes of some interfering metals, e.g. nickel and copper decompose in a graphite furnace at 400 °C [20].

For removal of interferents rinsing of column with a solvent solution before elution step is often recommended. Rinsing the IIP column with 4 mL of water or 0.05 mol L^−1^ acetic acid caused the removal of 6.2 or 8.5 % of retained Ru(III), respectively. More than 24 % of the analyte was removed in this step from the CP.

**Fig. 3:**
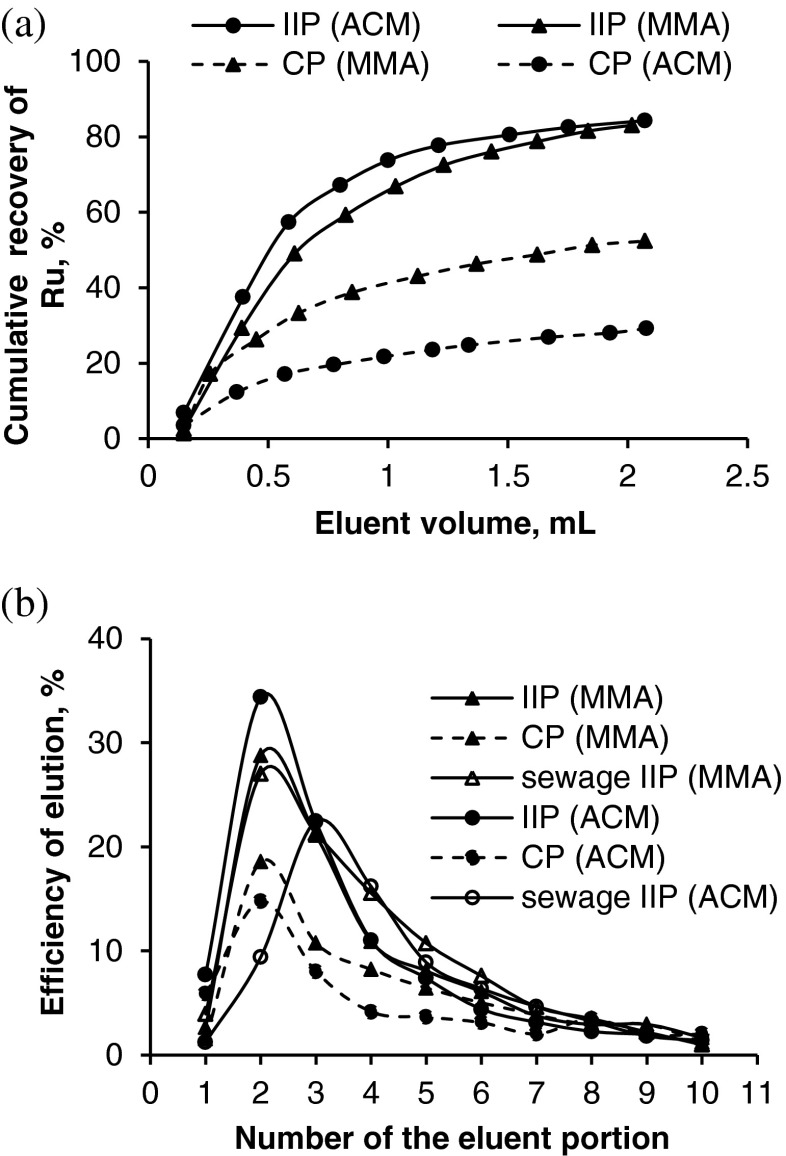
Elution of Ru(III) from IIP and CP with 0.2 mL portions of 4 mol L^−1^ NH_3_·H_2_O (eluent flow rate 0.4 mL min^−1^): **a** cumulative recovery of Ru(III) from standard solution (MMA – methacrylic acid, ACM – acrylamide), **b** elution profiles of Ru(III) from standard solution and sewage

Under optimized conditions the efficiency of the retention of ruthenium (100 ng, *n* = 3) on the Ru-TBA-MAA polymer was 93.4 ± 1.5 %, whereas the elution efficiency was 89.7 ± 0.7 %. The recovery of the analyte from the IIP, calculated as a ratio of the mass of Ru eluted from the column to the initial mass of Ru loaded onto the column, was 83.8 ± 1.8 %, while from the CP was 45.1 ± 5.9 %. The efficiency of the retention of ruthenium on the Ru-TBA-ACM polymer was 89.0 ± 0.7 %, whereas the efficiency of elution was 93.9 ± 2.9 %, and the recovery was 83.6 ± 2.8 %. The recovery of ruthenium from the CP was 30.2 ± 3.1 % (Fig. 3a). Reproducibility of the results for 15 successive sorption–desorption cycles was good (RSD < 7 %), allowing multiple use of the sorbent as column filling in flow procedures.

The breakthrough capacity of the polymer was determined according to the procedure described in [13]. The capacity of the polymer prepared with methacrylic acid was 36.6 μg g^−1^ for the IIP and 25.1 μg g^−1^ for the CP. The capacity of this IIP is about 16 times larger than the capacity of the Ru(III)-allyl acetoacetate imprinted polymer [14]. The capacity of the polymer prepared with acrylamide was 34.5 μg g^−1^ for the IIP and 21.0 μg g^−1^ for the CP. The average durability of the column filled with IIP was around 300 analytical sorption/desorption cycles for the Ru-TBA-MMA polymer and 250 cycles for the Ru-TBA-ACM polymer.

These results indicate minor influence of the type of functional monomer on the analytical performance of polymer, however it seems that the affinity of ruthenium for the Ru-TBA-MMA polymer is slightly higher. The profiles of elution shown in Fig. 3b confirm that Ru(III) is more specifically bound to the IIP than to the CP, and that there is a slight difference in the affinity of both polymers for Ru(III). Owing to better characteristics of the Ru-TBA-MMA polymer, this polymer was used for interference studies and analytical applications.

### Study of interference effects

The imprinting effect and the ion-recognizing behavior of imprinted materials are reflected by polymer selectivity in the presence of competing ions. The selection of metal ions for this study was based on the similarity of chemical properties of other metals to the analyte, the similarity of an ionic radius, and a degree of interference effect in the determination of Ru by ETAAS technique. The most serious interference in the ruthenium determination is caused by the presence of Co(II), Ni(II), and Pt(IV), Pd(II) and Rh(III) ions [15].

The distribution ratios (*D*, mL g^−1^), selectivity coefficients (*α*) and relative selectivity coefficients *(α*_*r*_) of Ru(III), with respect to Pd(II), Pt(IV), Rh(III), Co(II), Ni(II), and Fe(III) ions determined for the IIP were calculated in a dynamic system from the equations described in [15]. A comparison of *D* values for Ru(III) ions on the IIP and CP polymers shows that higher distribution ratios were achieved on the imprinted polymer (Table 1). These values are higher for Ru(III) than for other studied ions with the exception of Ni(II). The high *D* values obtained for Ni(II) ions both on IIP and CP suggest nonspecific sorption of these ions to the polymeric network. The relative selectivity coefficients, which express the selectivity of the imprinted polymers in comparison to the control polymer, are greater than 2.2. The selectivity of the polymer studied in this work is much better than the selectivity obtained for polymers based on imprinted complexes of Ru(III) with thiosemicarbazide [15], but slightly worse than that obtained for polymers with imprinted Ru(III)-benzaldehyde thiosemicarbazone complex [13].

**Table 1:** Selectivity parameters of IIP and CP polymers with methacrylic acid for Ru(III) ions against competitive sorption of other metal ions (2 mL of sample: 100 ng Ru + 100 ng of other ion, pH 4.1, flow rate: 1.2 mL min^−1^, n = 3)

Metal ion	Distribution ratio (D), mL g^−1^		Selectivity coefficient, *α*		*α*_r_
	CP	IIP	CP	IIP	
Ru(III)	60.9	283	–	–	–
Pt(IV)	2.36	1.97	25.8	144	5.6
Pd(II)	186	99.6	0.30	2.80	9.5
Rh(III)	1.80	1.44	33.8	196	5.8
Fe(III)	53.9	103	1.13	2.70	2.2
Co(II)	157	102	0.39	2.80	6.9
Ni(II)	439	466	0.14	0.61	4.3

The effect of interfering ions on the retention of ruthenium and its elution was also assessed. The efficiency of retention of Ru(III) on the IIP from a solution containing up to 1 μg of other metal ions was slightly affected by the presence of Pt(IV) (Table 2). The efficiency of elution of Ru(III) from the sorbent and in consequence its recovery was influenced only by the presence of Fe(III) ions. At solutions of pH > 4 higher amounts of Fe(III) ions may precipitate in the form of Fe(OH)_3_. During the course of elution with ammonia the ammonia complexes of Ru(III) (e.g. [Ru(NH_3_)_3_]^3+^) may possibly be sorbed on the surface of formed iron hydroxide precipitate and slowly undergo decomposition as it was observed for other platinum group metals [20]. In order to remove Fe(III) ions from the column, the sorbent was cleaned-up with 4 mL of 0.05 mol L^−1^ acetic acid before elution of the analyte was performed. During the clean-up a small part of the analyte (8.5 %) was removed. As a consequence the recovery of analyte in the presence of 10-fold excess of Fe(III) was lower by 17 % when compared to the recovery obtained for a standard solution. Better results were obtained when the sample pH was adjusted to 3.5, which prevented the formation of Fe(OH)_3_ precipitate [21]. The recovery calculated from the calibration graph prepared under the same conditions was lower only by 7 %. The extra clean-up of the column with 0.05 mol L^−1^ acetic acid didn’t affect the analyte recovery. These results confirm that the IIP can be used as a solid sorbent for separation of trace amounts of Ru(III) from other ions.

**Table 2:** Effect of the presence of competitive ions on the separation of Ru(III) ions (100 ng) on IIP with methacrylic acid (2 mL of sample: pH 4.1, mean value ± SD for *n* = 3)

Metal ion	C_int_/C_Ru_	Ru retention efficiency, %	Ru elution efficiency, %	Recovery,%
Ru(III)	–	93.4 ± 1.5	89.7 ± 0.7	83.8 ± 1.6
Pd(II)	1	91.2 ± 1.3	91.6 ± 2.1	83.6 ± 2.1
	10	86.9 ± 3.2	91.3 ± 2.3	79.3 ± 4.8
Pt(IV)	1	87.8 ± 2.0	91.2 ± 0.9	80.1 ± 1.3
	5	91.5 ± 2.2	91.3 ± 2.3	83.6 ± 4.1
	10	74.7 ± 3.2	94.8 ± 1.9	72.7 ± 1.3
Rh(III)	1	92.7 ± 3.5	89.9 ± 1.4	83.3 ± 2.1
	10	83.2 ± 1.9	91.4 ± 3.0	76.0 ± 3.3
Co(II)	1	94.5 ± 2.9	90.9 ± 2.9	85.9 ± 1.0
	10	93.1 ± 3.7	94.3 ± 1.7	87.6 ± 3.0
Ni(II)	1	96.7 ± 4.2	91.7 ± 3.9	88.7 ± 6.4
	10	83.5 ± 1.8	98.5 ± 4.7	82.2 ± 2.3
Fe(III)	1	96.6 ± 1.8	84.6 ± 1.9	81.8 ± 2.8
	10	97.7 ± 0.4	48.9 ± 4.6	47.8 ± 4.5
	10	97.8 ± 2.1	68.3 ± 1.9	66.8 ± 1.5^a^
	10	95.2 ± 1.1	77.6 ± 6.6	76.9 ± 0.6^b^
	10	94.4 ± 0.8	79.8 ± 2.4	75.4 ± 2.8^c^

^a^ Column clean-up with 4 mL of 0.05 mol L^−1^ CH_3_COOH,

^b^ Sample pH 3.5,

^c^ Sample pH 3.5 and column clean-up with 4 mL of 0.05 mol L^−1^ CH_3_COOH.

### Analysis of environmental samples

For the determination of ruthenium by ETAAS the calibration graph technique was used. The standard ruthenium solutions were submitted to the separation procedure on IIP using 2 mL of the eluent solution. The calibration graph was linear up to 16 ng mL^−1^ of Ru. The limit of detection of Ru (calculated as LOD = blank + 3SD_blank_ according to IUPAC recommendation) was 0.21 ng mL^−1^ for 10 mL sample, while the limit of quantification (LOQ = blank + 10SD_blank_) was 0.77 ng mL^−1^. The limit of detection is lower than that obtained by means of ion-exchange resins and inductively coupled plasma optical emission spectrometry [7, 10] or inductively coupled plasma mass spectrometry [5, 7, 22] (Table 3). The enhancement factor, defined as the ratio of the slope of the calibration graphs before and after the preconcentration process, was 3.3.

**Table 3:** Dynamic solid phase extraction procedures for separation/preconcentration of ruthenium from environmental samples

Type of sorbent	Optimal preconcentration/separation conditions		Comment	Detection technique; LOD	Analysed samples	Ref.
	Sorption	Elution				
Epoxy-imidazole resin	Mass of sorbent: 0.1 g Sample: pH 4 FR: 2 mL min^−1^	Eluent: 0.16 mol L^−1^ TU + 6 mol L^−1^ HCl (16 mL) FR: 1.5 mL min^−1^	Precision as RSD: <5 % Interferences: Ni(II) > 2 μg mL^−1^	ICP OES; 10–100 ng mL^−1^	Metal smelter	[10]
Cation exchange resin Amberlite CG-120	Volume of sorbent: 3 mL Conditioning agent: 6 mol L^−1^ HCl (20 mL), 0.2 mol L^−1^ HCl (15 mL) Sample: 0.2 mol L^−1^ HCl	Eluent: 0.2 mol L^−1^ HCl (4 mL)	Precision as RSD: < 8 %	ICP MS; < 4 ng g^−1^	Copper-nickel sulphide ores	[22]
Anion-exchange resin AG 1-X8	Mass of sorbent: 1.0 g Conditioning agent: 6 mol L^−1^ HNO_3_, HCl, H_2_O Sample: 0.1 mol L^−1^ HCl FR: 1 mL min^−1^ Clean-up: 0.1 mol L^−1^ HCl (12 mL)	Eluent: 5 mol L^−1^ HCl + 5 mol L^−1^ HClO_4_ (12 mL) FR: 0.5 – 1 mL min^−1^	–	ID ICP MS; 0.41 ng g^−1^	Peridotite (CRM: WPR-1, WMS-1), Meteorite sample (Orgueil)	[5]
Anion-exchange resin Dowex 1-X8	Mass of sorbent: 1 g Sample: 0.4 mol L^−1^ HCl FR: 2 mL min^−1^ Clean-up: 0.1 mol L^−1^ HCl (10 mL)	Resin was ashed at 550 °C, dissolved with 1 mL of conc. HCl and HNO_3_	–	USN ICP MS; pg g^−1^	Geological samples, CRM: WMG-1, WITS-1	[8]
Anion-exchange resin Dowex 1-X8	Conditioning agent: 1.0 mol L^−1^ HCl (100 mL) Sample: 1 mol L^−1^ HCl FR: 2 mL min^−1^	Eluent I: 0.3 mol L^−1^ TU in 0.1 mol L^−1^ HCl (75 mL) Eluent II:12 mol L^−1^ HCl (100 mL)	Precision as RSD: 16.6–57.9 %	ICP MS; 0.22 ng mL^−1^ ICP OES; 14 ng mL^−1^	Geological reference materials: PTM-1, PTC-1, SARM 7	[7]
IIP: Ru-TSd IIP: Ru-AcTSn	Mass of sorbent: 0.1 g Conditioning agent: 0.1 mol L^−1^ HCl (3 mL) Sample: pH 7.5; FR: 1.5 mL min^−1^ Clean-up: 0.05 mol L^−1^ acetic acid (2 mL)	Eluent: 0.2 mol L^−1^ TU in 0.2 mol L^−1^ HCl (3 mL) FR: 0.8 mL min^−1^	Precision as RSD: < 8.1 % Interferences: Pb(II) > 0.5 μg mL^−1^ Preconcentration factor: 10–25 Durability of sorbents: 100 cycles	ETAAS; 0.16 ng mL^−1^ 0.25 ng mL^−1^	Tap and river water, sewage, grass, hair	[15]
IIP: Ru-AAA	Mass of sorbent: 0.2 g Conditioning agent: 0.1 mol L^−1^ HCl (3 mL) Sample: pH 6.5; FR: 1 mL min^−1^	Eluent: 0.3 mol L^−1^ TU in 0.1 mol L^−1^ HCl (3 mL) FR: 1 mL min^−1^	Precision as RSD: < 3.5 % Preconcentration factor: 30 Durability of sorbents: 75 cycles	ETAAS; 0.32 ng mL^−1^	Tap and river water, municipal and road sewage, grass	[14]
IIP: Ru-BnTSn	Mass of sorbent: 0.1 g, Conditioning agent: H_2_O (4 mL) Sample: pH 8; FR: 0.6 mL min^−1^	Eluent: 0.3 mol L^−1^ TU in 0.3 mol L^−1^ HCl (2 mL) FR: 0.2 mL min^−1^	Interferences: Rh(III), Cd(II) > 5 μg mL^−1^ Precision as RSD: < 6.6 % Preconcentration factor: 25 Durability of sorbents: 40 cycles	ETAAS; 0.26 ng mL^−1^	Tap and river water, municipal sewage, road runoff, grass	[13]
IIP: Ru-TBA	Mass of sorbent: 0.1 g Conditioning agent: H_2_O (4.5 mL) Sample: pH 3.5 FR: 1.2 mL min^−1^ Clean-up: 0.05 mol L^−1^ acetic acid (4 mL)	Eluent: 4 mol L^−1^ NH_3_ (2 mL) FR: 0.4 mL min^−1^	Interferences: Pt(IV), Fe(III) > 5 μg mL^−1^ Precision as RSD: < 6.4 % Preconcentration factor: 40 Durability of sorbents: 300 cycles	ETAAS; 0.21 ng mL^−1^	Tap and river water, municipal sewage, road runoff, road dust, CRM: SARM 76	This paper

*ETAAS* electrothermal atomic absorption spectrometry, *ICP MS* inductively coupled plasma mass spectrometry, *ICP OES* inductively coupled plasma optical emission spectrometry, *ID* isotope dilution, *USN* ultrasonic nebulization, *TSd* thiosemicarbazide, *AcTSn* acetaldehyde thiosemicarbazone, *BnTSn* benzaldehyde thiosemicarbazone, *TBA* 2-thiobarbituric acid, *TU* thiourea, *CRM* certified reference material, *LOD* limit of detection, *FR* flow rate

The developed method was applied for separation/preconcentration of Ru from river and tap water, waste (municipal sewage, road runoff) and road dust spiked with analyte. Since ruthenium belongs to metals characterised by a slow rate of ligand exchange, the samples (spiked with 10–100 ng of Ru) were left overnight to reach equilibrium. The recovery of analyte from river water, tap water and municipal sewage was in the range of 97.1–99.7 %. The reproducibility of results expressed as RSD was in the range of 1.1–4.1 % (Table 4). The profile of elution of ruthenium present in municipal sewage from the IIP (Fig. 3b) confirms a proper selectivity of the IIP. However, the recovery of ruthenium from road runoff was too high indicating the occurrence of matrix interferences. Rinsing of a column with 2 mL of MQ water efficiently removed excess of matrix from the sorbent (Table 4).

**Table 4:** Recovery of Ru(III) from various samples after its separation on IIP with methacrylic acid (2 mL of sample: pH 4.1, mean value ± SD, *n* = 3)

	V, mL	C_Ru_, ng mL^−1^	Ru added, ng	Recovery,%
MQ water	2	50	100	98.7 ± 2.8
River water	10	1	10	97.1 ± 1.7
	20	1	20	95.7 ± 3.4
	60	1	60	93.1 ± 0.7
	80	1	80	89.8 ± 1.3
	100	1	100	80.9 ± 4.9
	2	50	100	97.1 ± 4.1
Tap water	2	50	100	99.7 ± 2.2
Municipal sewage	2	50	100	98.4 ± 2.1
Road runoff	2	50	100	94.5 ± 2.8^a^
Road dust	6	16.7	100	90.0 ± 3.3^b^
CRM SARM 76	2	–	–	97.9 ± 5.6^b,c^

^a^Column clean-up with 2 mL of MQ water,

^b^Sample pH 3.5, column clean-up with 4 mL of 0.05 mol L^−1^ CH_3_COOH,

^c^Against the reference value (0.49 ± 0.023 μg g^−1^)

Slightly modified procedure (sample pH adjusted to 3.5 and column clean-up with 0.05 mol L^−1^ CH_3_COOH) was used for the determination of Ru in road dust and platinum ore (CRM). Because the content of Ru in road dust was below the limit of detection of the method, the known amount of analyte was added to a mineralized sample, then the sample underwent the pretreatment procedure. The recovery of Ru from road dust was 90.0 ± 3.3 %. The good accuracy of the method was confirmed by analysis of certified reference material of platinum ore (SARM 76) (Table 4). The signals of ruthenium registered directly for the solution of digested CRM sample and after the separation procedure (not shown) reveal efficient elimination of matrix interferences on the used IIP sorbent.

The ability of the prepared polymers to preconcentrate trace amounts of Ru(III) ions (1 ng mL^−1^) from large volumes of river water (10–100 mL) was also tested. The results confirmed that the procedure may be applied to the separation of trace amounts of Ru(III) from volumes up to 80 mL (Table 4).

## Conclusions

New selective sorbents for the separation of Ru(III) by the SPE technique were prepared by an ion imprinting technique using complex of Ru(III) with 2-thiobarbituric acid (template), and methacrylic acid and acrylamide as functional monomers. It was found that the type of functional monomer only slightly affected the sorption properties of the sorbent. However, the affinity of Ru(III) for the Ru-TBA-ACM polymer was to some extent weaker than for the Ru-TBA-MMA polymer. The Ru-TBA-ACM polymer was characterised also by a lower surface area, sorption capacity and shorter durability. Thus, the Ru-TBA-MMA was used for interference studies and analytical application. This IIP is characterized by greater selectivity and adsorption capacity in comparison with CP. The developed SPE method can be applied in a wide range of sample pH (3.5–10), which promotes the analysis of various types of samples. Acidic sample pH allows the selective separation of Ru from constituents of digested environmental samples, whereas neutral pH can be an advantage during separation of analytes from natural waters and wastes. The procedure of ruthenium separation on IIP from interfering matrix is shorter, more universal and selective in comparison to others (Table 3). The developed method is suitable for determination of trace amounts of ruthenium in complex environmental samples.
